# MARKERS AND BIOMARKERS OF DEMENTIA BDNF GENOTYPE AND CHANGES IN WHITE MATTER HYPERINTENSITIES AND HIPPOCAMPAL MICROSTRUCTURE IN OLDER MEN AND WOMEN

**DOI:** 10.1093/geroni/igz038.2172

**Published:** 2019-11-08

**Authors:** C Elizabeth Shaaban, Andrea Metti, Cindy Barha, Kristine Yaffe, Caterina Rosano

**Affiliations:** 1 University of Pittsburgh Graduate School of Public Health, Pittsburgh, Pennsylvania, United States; 2 University of British Columbia, Vancouver, British Columbia, Canada; 3 UCSF School of Medicine, San Francisco, California, United States; 4 University of Pittsburgh, PIttsburgh, Pennsylvania, United States

## Abstract

Brain-derived neurotrophic factor (BDNF) may protect against cerebral gray and white matter impairments in older age. The val66met genetic polymorphism of BDNF is recently emerging as an early marker of brain structural integrity. However, evidence is sparse, cross-sectional, and mostly in men. In a longitudinal cohort study of community-dwelling older adults (N=139, mean age=81.6, 58% female, 58% white, mean follow-up=3.4 years), we estimated the overall and sex-stratified effects of BDNF val66met polymorphism on changes in cognition and gray and white matter macro- and micro-structure. Annualized percent change was computed for volume of white matter (WM) hyperintensities and gray matter (GM), fractional anisotropy of normal appearing WM, and mean diffusivity (MD) of GM in whole brain and memory and executive control function networks. Significant associations were adjusted for variables differing by genotype (race, APOE4, diabetes, triglycerides, smoking). Compared to met carriers, val homozygotes had slower annual whole brain WMH accrual (median (IQR) 31.4% (61.7) vs. 60.7% (92.4)), stronger in women. Met carriers had slower annual accrual of hippocampal MD (median (IQR) 1.26% (0.92) vs. 1.85% (1.09) for right hippocampus, stronger for women, and 1.45% (1.06) vs. 1.97% (1.22) for left hippocampus, stronger for men) compared to val homozygotes. Associations were robust to covariates’ adjustment. BDNF polymorphism was not associated with cognitive changes. BDNF polymorphism may help in early identification of those more likely to resist accrual of WMH and loss of hippocampal microstructural integrity, with effects varying by sex.

